# Async or Askew: The Chat Cascade and Clinical and Educational Disruption

**DOI:** 10.1007/s11606-026-10648-4

**Published:** 2026-07-21

**Authors:** David Alexander Goodson, Brittany Garcia, Michael Hogarth, Shin-Ping Tu

**Affiliations:** 1 Department of Internal Medicine, University of California, Sacramento, CA USA; 2 Division of Bioinformatics, Department of Medicine, University of California, San Diego, CA USA

**Keywords:** communication, secure chat, electronic messaging, medical education, inpatient medicine

## INTRODUCTION

Since the rapid adoption of secure electronic messaging (SEM) across healthcare systems in the USA, few studies have examined its impact on healthcare teams and patients. Though surveys of SEM users indicate favorable reception, we are only beginning to understand its role in clinical medicine.^1,2^ We present the case of a multi-visit patient on his third admission within the month and assess the messaging involved in his care and discharge planning. We highlight the volume of electronic communication necessary in the care of complex patients, its potential impact on trainees, and the need for further research on this mode of communication.

### CASE

A 64-year-old man with multiple comorbidities and housing insecurity presented to the Emergency Department with a chief complaint of chest pain 1 h after leaving against medical advice from an admission for acute renal failure. His past medical history included chronic obstructive pulmonary disease, coronary artery disease, congestive heart failure, atrial fibrillation, and chronic kidney disease. His mental health history included bipolar disorder, personality disorder, major depression, and alcohol use disorder. After a negative evaluation for life-threatening causes of chest pain, the patient was admitted for management of urinary retention, acute renal failure, and his comorbidities. A urinary catheter was placed, and his renal failure improved over the subsequent days.

The acute medical issues were addressed, and the patient was quickly ready for discharge. However, given his many comorbidities, from the longitudinal treatment standpoint, his care presented new challenges to his inpatient team. In the preceding year, he had more than 50 Emergency Department encounters and 25 hospitalizations. Anticipating this complexity, the primary team consulted psychiatry, discharge planning (DCP), and urology to develop a comprehensive care plan.

Psychiatry determined the patient possessed neither decisional nor dispositional capacity (the ability to care for himself after discharge). It became a challenge to find him a semi-monitored, semi-supported environment, and further delayed his discharge.

Urology agreed to perform a Holmium Laser Enucleation of the Prostate (HOLEP) as definitive management of his chronic urinary retention due to benign prostatic hypertrophy (BPH). The internal medicine and urology teams both felt this was a superior option because of concerns about medication noncompliance. His hospitalization was prolonged while waiting for his procedure, first to identify a surrogate decision maker, then due to operating room availability.

After a 15-day hospitalization, on the day after completing his procedure, the patient was discharged to a semi-monitored environment. However, despite a striking volume of electronic messaging involved in the care of this patient, a communication breakdown did occur, and his oxygen was delivered to the wrong address.

To understand the complexity and scope of secure electronic messaging in this patient’s care, as well as its potential impact on his medical team, we reviewed all related messages during his hospitalization and summarized our observations below.

## DISCUSSION

Secure electronic messaging (SEM) can facilitate the movement of clinical information quickly, without necessitating in-person communication or the time investment to return a page.^3^ However, unlike verbal communication, which allows maximal information transfer, planning, and clarification immediately, the short form (compact) text and asynchrony of SEM might engender ambiguity that persists until clarification.^4,5^ Additionally, the potential for a “fire-and-forget” communication style with minimal sender effort, implicit expectations of rapid responses, and no sender awareness of recipient readiness for information risks facilitating distraction and task-interruption.^6^ As the use of SEM proliferates, it is imperative to take stock of its potential for positive and negative impacts.

The care of this patient encompassed the movement of a large amount of communication across a broad interdisciplinary team (Table 1). His care team comprised 107 members across 14 different roles and included residents and attendings from internal medicine, emergency medicine, psychiatry, and urology, but no medical students. This team was involved in 128 secure electronic messaging threads (SEMT) with a total of 1145 messages sent within 163 conversations over 15 days. Here, “conversations” are defined by sent messages that receive replies, whereas SEMT are running chats between team members that contain one or more conversation. On average, the daily volume of messages sent regarding this patient was 76, which includes a daily average of 11 new conversations among 12 individuals.

**Table 1:** Characterizing Message Frequency and Conversation Duration

	Total	Primary contact, IM resident^*^	All other IM residents^*^	Non-IM residents^*^	All attendings	Registered nurses	Discharge planning team
Daily messages							
Mean^†^	76.3	51.4	19.8	10.5	12.7	20.6	23.4
Median^†^	67	33	13	9	7	12	12
Min–max messages	36–158	26–126	2–84	1–23	1–46	1–89	1–83
Days involved	15	12^‡^	15	14^§^	4	15	9
Conversation duration (minutes)							
Mean^†^	37.3	46.2	39.1	115	45.46	40.7	94.1
Median^†^	13.6	16.9	11.3	70	29	14	38.7
Min–max minutes	0.3–315	0.5–262	0.3–262	0.9–373	0.6–182	0.3–315	2.4–259
Total conversations	163	68	72	27	8	113	16

^*^IM= internal medicine, non-IM residents are from urology, emergency medicine, and psychiatry

^†^Mean and median for “Daily Messages” are per person per day and for “Conversation Duration” are per conversation

^‡^The primary contact was covered by senior residents on their days off service

^§^Emergency medicine, 1 day; urology, 10 days; psychiatry, 6 days. Some days overlap

Although this vignette likely exemplifies an extreme of SEM communication due to this patient’s medical, social, and behavioral complexity, it raises the questions of whether SEM, as currently applied, can engender an undue chat burden on trainees, excess information communication, deferred decision-making, or shifting time demands of clinical practice.

Electronic communication modalities are likely experienced differently across a patient’s medical team. For example, in this case, the average volume of messages sent or received daily by an individual varied by their clinical role (Table 1). In particular, the time and communication burden for clinical and dispositional coordination was borne primarily by the patient’s primary inpatient care team—specifically, the internal medicine trainees.^5^ This trend is consistent with prior analyses finding the daily average SEM message volume of trainees to be approximately 1.17–1.5 and 2.45–4.9 times greater than that of attending physicians and registered nurses, respectively.^2,7^ This may result from increased reliance on messaging (instead of documentation) for care plan clarification,^8^ increased solicitation of secondary opinions by residents,^1^ or the observation that, as lower-hierarchy team members and accessible first-line communicators, trainees are central to inpatient communication networks.^5^

Still, the degree of trainee messaging in this case is remarkable. In a similar analysis at another academic medical center, hospitalists sent or received an average of 11 messages per patient per day, for a total of 130 messages per day.^9^ By comparison, the intern serving as Primary Contact for this patient sent or received an average of 51 messages per day, more than twice the daily messaging volume experienced by other IM trainees, non-IM trainees, and RNs.

While little guidance on the prudent use of secure messaging exists, the educational opportunity of caring for psychosocially and behaviorally complex patients should be balanced with the possible imposition of unlimited electronic communication. For example, 42% of messages were sent between 6:00 AM and 12:00 PM, 35% between 12:00 PM and 6:00 PM, and 23% from 6:00 PM to 6:00 AM. Not only can the volume of messages and burden of coordination for complex patients be particularly high, but a disproportionate amount of messaging occurs during morning rounds and may be dedicated to managing behavioral challenges (Table 2). Furthermore, trainees may be particularly susceptible to the time effects of ballooning text conversations.^10,11^ As SEM defines an era of electronic communication in medicine and shapes medical education, guidelines for the use of this communication modality are needed.^12^

**Table 2:** Secure Electronic Messaging Conversation Content^*^

Total conversations	163 (%)
Clinical information and care coordination	
Clinical information only	36 (22)
Clinical information and non-clinical information	36 (22)
Care coordination	16 (10)
Psychosocial and behavioral^†^	
Psychosocial and behavioral information only	42 (26)
Psychosocial and behavioral and non-psychosocial and behavioral information	41 (25)
Requests for pain medications	26 (16)
Discharge planning	
Discharge planning only	21 (13)
Discharge planning and non-discharge planning information	10 (6)

^*^Categories are not mutually exclusive; conversations often cover more than one type of content

^†^Psychosocial and behavioral care are broken out as a subcategory of clinical care

Despite the proliferation of electronic communication modalities, few studies have evaluated the content communicated or its impact on care.^3,13^ In this case, a striking portion of conversations communicated information that—while important in its own right—would not change care. While just over half of the conversations communicated clinical or care-coordination information, such as the results of a bladder scan or when imaging would be completed, approximately half of the conversations pertained to the management of psychosocial and behavioral challenges, such as requests to leave (despite lacking capacity), the need for a new IV (after the patient removed the last), or requests for opioid medications (though not indicated) (note, conversations often communicate more than one type of information). Additionally, fifty-four individual messages (4.7%) related to managing patient requests for IV hydromorphone and an additional 53 (4.6%) related to managing requests for pain medications generally. While it is difficult to categorize any communication as redundant or low-value, it is hard to imagine pager-based systems of communication with as many unique pages for IV opioid use, Foley discontinuation, or self-discharge. Where SEM has lowered barriers to remote communication, it may also have lowered the bar for what is communicated.

Methods for evaluating what information is communicated—and its clinical significance—could facilitate the development of guidelines for best practices and analyses of the impact of SEM on teamwork in patient care. For example, the degree of communication regarding behavior in this case raises the question of how much bedside management of patient behavior is shifted to physician trainees. A similar concern was raised in a recent JAMA Internal Medicine Inside Story (2025) by Kochar, Knees, and Arnold that questioned whether secure messaging is undermining trainees’ knowledge and autonomy by allowing them to defer decision-making to specialists or attendings.^14^ If secure messaging facilitates deferring decision making to the next highest license or training—nurses deferring to doctors, trainees to their attendings, or internists to subspecialists—clinicians may shift their practices to the lower ends of their licenses. Communication that undermines individual competency without demonstrating improved patient safety may ultimately undermine efficiency, fragment care, and accountability and yield lower quality care in the long run.

Finally, delineating SEM conversations highlights the amount of time clinicians spend engaging with this modality of patient care. For example, for this one patient, we found an average of 11 new conversations per day lasting a median 14 min, which requires a daily average of 2.6 h of healthcare team cognition. This estimate may be higher on days involving discharge planning, consultant engagement, or coordination of more complex operations, such as obtaining an MRI, as such conversations can span hours (Table 1). Moreover, we also find the median time between sent messages in a SEM conversation, at 48 s, to be short.^9^ This underscores the concern that short time intervals between messages may increase task switching, either away from the task at hand to answer messages or to address new tasks. Indeed, how would clinicians care for other patients during these 2.6 h without repeatedly switching tasks?

According to cognitive load theory, unfiltered interruptions, multitasking, and ongoing conversations may lower a physician’s cognitive performance.^15^ This could be catastrophic during the management of high-risk orders or when clinical care demands critical thinking. Studies are beginning to explore the potential burden of unlimited messaging and its impact.^10,11,16^ As inpatient clinicians increasingly manage patients and engage their teams through screens, it will be important to understand what unique opportunities and challenges arise.

Several barriers will need to be addressed in order for research to delineate how secure messaging can best be leveraged to enhance patient care. SEM data is often deleted after a set amount of time, as at our own institution, limiting opportunities for retrospective analysis and hypothesis generation.^5,7,17,18^ SEM design and use are idiosyncratic to healthcare systems and individuals so that even quantitative analyses may not standardize easily. Classifying SEM content involves subjectivity, which should be acknowledged. SEM is unable to capture communication occurring in-person or by telephone. Finally, theory-based and empirically grounded frameworks are needed to support critical evaluations of SEM’s role in patient care and healthcare teamwork.

In conclusion, secure messages and their content remain a rich source of data that is underutilized in evaluating this new tool’s impact on medical education, team communication, and physicians’ time and cognitive work. Methods for quickly and accurately categorizing content should be developed. Previously, such an analysis was limited by reliance on complicated natural language pipelines or, as in our case, manual categorization. However, the emergence of HIPAA-compliant large language models has significantly reduced the barrier to text analysis and could be employed here. This data would allow future studies to analyze messages at scale and link secure electronic message communications to actions taken and patient outcomes.
